# Pregnancy After Laparoscopic Hysteropexy: A Systematic Review

**DOI:** 10.3390/jcm14082777

**Published:** 2025-04-17

**Authors:** Anna Pitsillidi, Laura Vona, Stefano Bettocchi, Sven Schiermeier, Günter Karl Noé

**Affiliations:** 1 Department of OB/GYN, Rheinland Klinikum Dormagen, Dr.-Geldmacher-Straße 20, 41540 Dormagen, Germany; guenter.noe@uni-wh.de; 2 Department of Medical and Surgical Sciences, Institute of Obstetrics and Gynaecology, University of Foggia, 71122 Foggia, Italy; laura.vona@unifg.it (L.V.); info@stefanobettocchi.com (S.B.); 3 Department of OB/GYN, University of Witten Herdecke, 58448 Witten, Germany; sven.schiermeier@uni-wh.de

**Keywords:** pregnancy, hysteropexy, prolapse, laparoscopy

## Abstract

**Background:** Nowadays, there is an increasing desire among women suffering from pelvic organ prolapse (POP) to choose a uterus-sparing surgical treatment in order to preserve their fertility. The objective of this study was to conduct a systematic review of the literature to assess how pregnancy and delivery affect the recurrence of POP in women who had previously undergone laparoscopic hysteropexy as well as to improve and individualise the future counselling of patients of reproductive age desiring uterine-preserving treatment for POP. **Methods**: A comprehensive literature review was conducted using the MEDLINE (PubMed), Web of Science, and Scopus databases for articles published until January 2025, without previous historical limits. The research strategy adopted included different combinations of the following terms: hysteropexy, pregnancy, laparoscopy, and prolapse. **Results**: A total of ten case reports and three case series met the inclusion criteria for the review, comprising 26 patients. All authors used laparoscopic sacral hysteropexy (LSHP) for the treatment of POP. All patients underwent caesarean delivery at a mean gestational age of 38 weeks. Over a mean follow-up period of 9 months, only 4% of patients developed a recurrent uterine prolapse. A total of 8% of the patients developed de novo anterior compartment prolapse, 8% developed a recurrence of anterior compartment prolapse, and 4% developed posterior compartment prolapse. **Conclusions**: LSHP seems to be a safe option for women of reproductive age with incomplete family planning, as it does not seem to negatively impact foetal growth. Pregnancy does not appear to affect the long-term efficacy of hysteropexy in maintaining apical support. Given the limited data on the safety and efficacy of uterine-sparing surgery for POP followed by a subsequent pregnancy, further evidence is of great importance towards evaluating safety, efficacy, and providing better counselling for women.

**Systematic review registration**: PROSPERO, CRD420250651516

## 1. Introduction

Pelvic organ prolapse (POP) is a frequent medical condition characterized by the protrusion or herniation of pelvic organs through the vaginal walls and pelvic floor [1]. It is a condition that impacts the lives of many women globally and significantly affects their quality of life [2]. Multiple risk factors such as parity, vaginal delivery (especially the first one), menopause, high Body Mass Index (BMI), or even comorbidities which increase the intrabdominal pressure contribute to the weakening of the pelvic floor connective tissue, leading to POP [3,4,5,6]. The estimated prevalence of POP is 3–6%, when based on the presence of symptoms. However, this figure increases to 50% when assessed through vaginal examination, which reflects anatomical changes without considering the symptoms or the severity of the prolapse [7]. Although POP is most common after menopause, it seems to also affect 10–26% of women of reproductive age [8].

Conservative treatment (such as pessaries and pelvic floor training) should be the first-line approach for patients with incomplete family planning, with surgical therapy being considered only as an alternative for those who do not experience any improvement [9,10,11]. However, nowadays, with the rising birth rate among older women, the likelihood of conception after surgical treatment for pelvic floor dysfunction is increasing [12]. Various uterus-preserving techniques—including abdominal, vaginal, and laparoscopic approaches—have been developed, demonstrating reduced operative times and lower estimated blood loss. Nevertheless, there is still no established consensus on the optimal method [13]. The literature on pregnancy and childbirth following hysteropexy is limited, with most available research consisting of case reports and case series. The lack of robust evidence on the safety and efficacy of uterine-preserving procedures for POP treatment in women who later conceive makes counselling challenging, particularly regarding how pregnancy and delivery mode may influence the risk of POP recurrence. Due to the absence of a consensus on managing pregnancy after pelvic floor reconstructive surgery, many obstetricians opt for caesarean section, while others view incomplete family planning as a relative contraindication for POP surgery, especially when mesh is used [14,15,16].

The objective of this study was to conduct a systematic review of the literature to assess how pregnancy and delivery affect the recurrence of POP in women who had previously undergone laparoscopic hysteropexy as well as to improve and individualize the future counselling of patients of reproductive age desiring uterine-preserving treatment for POP.

## 2. Materials and Methods

We performed our research on MEDLINE (PubMed), Web of Science, and Scopus databases and considered articles published until January 2025, without previous historical limits. The research strategy adopted included different combinations of the following terms: hysteropexy, pregnancy, laparoscopy, and prolapse.

**Eligibility Criteria**

Articles were included if they met the following PICO (population, intervention, comparison, outcome) criteria [17]:

- **Population (P)**: female patients who underwent laparoscopic surgery for POP with uterine preservation and subsequently conceived.
- **Intervention (I)**: hysteropexy with the use of a mesh.
- **Comparison (C)**: it was not relevant to use this component for the purpose of this systematic review.
- **Outcome (O)**: recurrence of apical prolapse or prolapse of another compartment and foetal growth outcomes.

For the article selection, we included studies that focused on pregnancy after laparoscopic hysteropexy and prolapse recurrence. Case reports, randomised controlled trials, prospective controlled studies, prospective cohort studies, retrospective studies, and case series were considered eligible. Reviews, letters to the editor, and abstracts accepted at conferences were excluded; however, relevant reviews were analysed to identify additional cases.

Ten case reports and three case series met all review requirements. All identified studies were examined for year, citation, title, authors, abstract, and full text. Duplicates were identified through manual screening performed by two researchers (LV and AP) and then removed.

**Study Selection and Quality Assessment**

The PRISMA guidelines were followed [17,18]. For the eligibility process, two authors (AP and LV) independently screened the titles and abstracts of all non-duplicated articles and excluded those not pertinent to the topic. The same authors independently reviewed the full texts of the remaining articles and identified those for inclusion. Any disagreements were resolved by consensus. Studies with ambiguous or insufficient data, low-quality data, or non-quantifiable outcomes were also excluded.

The methodological quality of the included studies was assessed using the JBI Critical Appraisal Checklist for case reports (Supplementary Table S1). This study has been registered in the PROSPERO database (registration number: CRD420250651516). The inclusion of only case reports in this review presents a risk of bias.

## 3. Results

**Figure 1:**
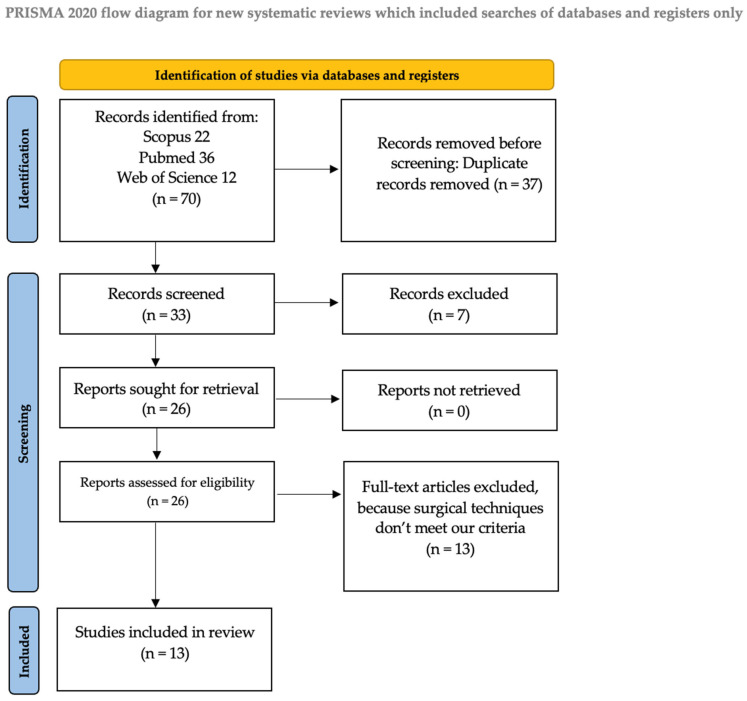
Flow diagram.

We identified 70 manuscripts; of these, 36 records were identified on PubMed, 12 were identified on Web of Science, and 22 were identified on Scopus. Records excluded for selection criteria and duplicates were n = 37. In our review, we included a total of 13 manuscripts and 27 patients at the end of the screening process (Table 1). The PRISMA flow diagram of the selection process is provided in Figure 1.

In our review, we examined the patients’ age, obstetrical history, POP-Q prolapse score, the surgical procedure and the Mesh used, uterine artery flow status during pregnancy, time interval between surgery and delivery, the gestational age at birth, the delivery modality, the postpartum follow-up, and occurrence of recurrence. We excluded from the review patients who underwent transabdominal and transvaginal procedures or surgical techniques that did not involve the use of mesh. Articles not relevant to the topic were also excluded.

In our analysis, the patients’ mean age was 35.7 years. One case report did not provide the patient’s age, and one study only mentioned an age range of 23–39 years. The mean parity of the patients was 2.6. Five studies reported complete staging data according to the Pelvic Organ Prolapse Quantification (POP-Q) system, and of these, the mean point C measurement was +1.6 cm. Five case reports mentioned only the POP-Q stage (II in four cases and III in two cases). Two studies did not report the uterine prolapse stage before and after pregnancy; however, it was noted that one patient experienced a relapse, while another one did not. The lack of any data for some studies and the use of different classifications could lead to interpretation bias.

All authors used laparoscopic sacrocolpopexy as the surgical technique for the treatment of apical prolapse. Regarding the type of mesh used, 89% of patients had a polypropylene mesh, 3.7% had a polyvinyl mesh, 3.7% a polypropylene and poliglecaprone mesh, and 3.7% a polyester mesh.

Only two authors investigated the uterine artery status during pregnancy. Among those, 19% of patients had normal uterine flow throughout the entire pregnancy, while 8% showed a significant right angulation of both uterine arteries at the level of the internal os at 23 weeks of gestational age; however, both the pulsatility index (PI) and resistance index (RI) remained within normal limits.

The mean interval between surgery and delivery was 17.2 months. One study did not provide this information, and another one reported only a range of 6 to 36 months. Regarding the delivery mode, 100% of the patients had a caesarean delivery at a mean gestational age of 38 weeks, although these data were not reported in two studies. One patient delivered at 39 weeks due to gestational diabetes and foetus in breech position.

The average weight of infants for whom the data were available was 3420 g, which is within the normal range for gestational age. In the mean follow-up time of 9 months, only 4% of patients developed a recurrent uterine prolapse. A total of 7.4% of the patients developed de novo anterior compartment prolapse, 7.4% developed a recurrence of anterior compartment prolapse, and 4% developed posterior compartment prolapse.

## 4. Discussion

Nowadays, there is a growing preference among women for uterus-sparing techniques for POP treatment. Several studies indicate that over one-third of patients would opt for uterine-preserving surgery if the outcomes were comparable to non-preserving procedures, while more than one-fifth would still choose to retain their uterus even if it is linked to less favourable results. There are many factors influencing this decision, including the desire to maintain fertility, the belief that the uterus plays a role in sexual function, and its significance in a woman’s sense of identity [32,33]. Several laparoscopic uterus-preserving techniques have been described for POP management, such as laparoscopic sacral hysteropexy (LSHP), laparoscopic hysteropectopexy (LHP), and laparoscopic lateral suspension (LLS). However, for the majority of these techniques, no pregnancy cases have been reported postoperatively [28].

Jefferis et al. reported six cases of pregnancy after laparoscopic hysteropexy with a polypropylene mesh wrapped around the cervix and fixated anteriorly on it and on the promontory (Oxford hysteropexy). All pregnancies resulted in a live birth, with birth weight on or above the 10th percentile. Regarding the delivery mode, all patients had deliveries by C-section and were shown to have a normally developed lower uterine segment. In only one case the mesh was reported to be over the lower uterine segment. The fixation of the mesh on the promontory was stable in all six patients. Postpartum follow-up showed no recurrence of apical prolapse, while two patients demonstrated a de novo cystocele [19]. A primary concern associated with this encirclage technique and a subsequent pregnancy was the potential compression of the uterine arteries by the mesh, resulting in compromising uterine blood flow leading to intrauterine growth restriction (IUGR). However, no PI or RI alterations were detected in the study population, while only one patient showed a right angulation of the uterine artery [19]. Rahmanou et al. also reported one case of a pregnancy after laparoscopy hysteropexy with the cervical encirclage method. Furthermore, Rahmanou et al. emphasized the monitoring of Doppler signals throughout the duration of the pregnancy. A right angulation of both uterine arteries was detected at 23 weeks of gestation, with the PI and RI remaining unaffected throughout the entire pregnancy. A C-section was also performed in this case [20]. Rahmanou et al. reported a good apical support with a de novo moderate anterior prolapse in the three-month postpartum examination, indicating that a full-term pregnancy can occur without complications after this procedure and that pregnancy does not adversely affect apical support in women who have undergone hysteropexy [20].

A retrospective cohort study examined 159 patients who underwent LSHP using a non-absorbable synthetic type one monofilament polypropylene mesh, fixated on the posterior part of the cervix at the level of the uterosacral ligaments and on the promontory. In this study population, eight patients conceived postoperatively. Seven of them delivered a full-term, healthy, normal weight infant with C-section, while one patient experienced a miscarriage in the first trimester and was treated medically. During the post-delivery evaluation, the recurrence of the apical prolapse was detected in only one of the patients [21]. No Doppler screening was needed in this study as the mesh was not wrapped around the cervix and did not influence the uterine blood flow. Furthermore, the placement of the mesh posteriorly on the cervix was shown to facilitate the uterine incision anteriorly during the C-section, as well as the management of early miscarriage [21].

Szymanowski et al. also reported a successful full-term pregnancy after LSHP combined with a modified Richardson’s and Burch colposuspension, terminated by a C-section. Both the postpartum vaginal examination and the quality of life (QOL) questionnaire demonstrated sufficient support in all levels of support as well as significant improvement regarding the overall well-being of the patient [22]. Another case of pregnancy post-LSHP was presented by Lewis et al. In this case, a lightweight polypropylene mesh was fixated on the anterior longitudinal ligament and the posterior side of cervix, leaving the anterior lower uterine segment free. At the 12-month postpartum evaluation, no recurrence of POP was detected. However, after 2 years, the patient experienced a POP recurrence of the anterior and posterior compartment [23]. Lewis et al. demonstrated in this trial that LSHP can be an effective and safe option for the treatment of POP in women desiring childbirth in the future, while also emphasizing the need for continued follow-up beyond the first postoperative year [23].

Gadonneix et al. reported another case of full-term pregnancy, which ended in an elective C-section at 38 weeks of gestation. A 48-month follow-up was carried out, revealing no POP relapse [24]. Furthermore, Albowitz et al. documented a subsequent pregnancy after LSHP, with a shorter re-evaluation period of three months, showing no signs of recurrence [25].

Algeri et al. also presented a patient who became pregnant after a laparoscopic hysteropexy with a “Y” propylene band fixated on the cervix and the promontory. Although this was an uneventful pregnancy, the patient experienced pelvic pain during the 32 weeks of gestation, was hospitalised at the 34th week, and delivered per C-section at the 35th week of gestational age. After the C-section, the pain diminished directly and her postpartum follow-up did not reveal any prolapse recurrence [27]. This study highlights that pelvic pain in a patient with a history of laparoscopic hysteropexy, especially in the third trimester, could be the result of the tension caused by the presence of the band and should definitely be considered in the differential diagnosis, alongside other possible explanations [27]. Furthermore, Busby et al. reported a pregnancy after LSHP, during which the patient also presented with pelvic pain in the 34th week of gestation, and underwent a C-section as a result. The apical support, two months after the delivery, was proven to be sufficient and the patient remained asymptomatic, one year later [30].

Rotem et al. retrospectively studied a case series of 25 patients who underwent LSHP with SERATEX^®^ SlimSling mesh^®^ for the treatment of apical prolapse. In this study population, four patients were reported to have conceived postoperatively. All of them delivered by C-section. Notably, no cases of prolapse recurrence were observed in these patients after pregnancy, with a median follow-up period of 65 months [29].

Samantray et al. and Pilka et al. also presented two patients who conceived after LSHP, delivered uneventfully via C-section, and were shown to have no POP relapse [26,28].

Another case of a full-term pregnancy following LSHP that ended in a C-section was also reported by Futcher et al. [31]. The patient was found to be free of recurrence during a follow-up period of two months postpartum [34].

As observed, data on pregnancy outcomes following laparoscopic hysteropexy are limited and primarily derived from small sample sizes of case reports or small case series. Therefore, limitations of this review include its retrospective nature, in which any data are missing, and hysteropectopexy was administered unblinded without a control group, which increases the risk of selection bias. Another major limitation is the significant heterogeneity among the studies in this review. Variations in preoperative prolapse severity, surgical techniques, definitions of success and failure, and the scope of reported data limit the ability to draw generalizable conclusions for the overall population. Lastly, many included studies have an insufficient follow-up time, making it challenging to accurately assess the long-term effects of pregnancy and childbirth on POP recurrence in women who had previously undergone laparoscopic hysteropexy.

Additional research is required to determine the optimal surgical method for managing apical prolapse in women of reproductive age with incomplete family planning. At present, there is a lack of comprehensive studies to validate the safety and efficacy of these techniques regarding a possible subsequent pregnancy or even the management of early pregnancy loss after hysteropexy.

It also remains unclear how the mesh continues to contribute in the support of the uterus during the pregnancy. One possible explanation could be that as pregnancy progresses, the enlarging uterus naturally supports itself within the pelvic cavity, thereby decreasing the tension on the mesh [21]. Furthermore, apart from the continuous mechanical remodelling of the uterus during pregnancy, biochemical changes in the connective tissue structures also contribute to the strengthening of the pelvic floor [34].

Nevertheless, the mesh used for the laparoscopic hysteropexy could potentially influence the uterine blood flow, which is necessary to support foetal growth, particularly by constricting the uterine arteries when it is positioned around the isthmus [35]. Doppler studies of the uterine arteries could be recommended to monitor such changes [28]. The fixation points of the mesh seem to also play an important role regarding the presence of pelvic pain during the pregnancy. Specifically, it has been suggested that the pain experienced during the third trimester may be due to the tension caused by the mesh, when it is fixated on the posterior side of the cervix [23,30].

Regarding the delivery mode, there is no consensus about the obstetrical management of women after laparoscopic hysteropexy. However, according to the existing literature, there seems to be a trend in favouring C-section in such cases, due to the unknown impact of a vaginal delivery on the surgical outcome and a possible recurrence of POP [25].

## 5. Conclusions

Concerning all subsequent pregnancy cases reported after laparoscopic hysteropexy, it is demonstrated that this procedure enables an uneventful, full-term pregnancy without influencing the support of the apical compartment. Although LSHP seems to be a promising, safe, and efficient technique, especially for women of childbearing age with incomplete family planning, there is an increasing need for more multicentre studies in order to identify the best therapeutic option for these patients and also enable a better and more detailed preoperative counselling.

## Figures and Tables

**Table 1:** Characteristics and data extracted of included studies.

Reference	Patients (n)	Age (Years) Mean	Parity, Mean	Prolapse Score POPQ System (Cervix—Point C cm) or POPQ Stage (I–IV) Prehyseropexy, Mean	Surgical Procedure- Mesh	Uterine Pulsatility Index (PI) and Resistance Index (RI)	Interval Between Surgery and Delivery (Months), Mean	Delivery Modality	Gestational Age (Weeks)/Birth. Mean	Birth Weight (g) Mean	Follow-Up After Pregnancy (Months) Mean	Recurrence of Apical Prolapse Postdelivery (n)
Jefferis H. et al. [19]	6	36.5	2.3	0.5	Sacrohysteropexy— Type 1 Polypropylene mesh	Normal In one patient uterine artery Doppler at 23 weeks showed significant right angulation of both arteries at the level of the internal os but PI and RI remained within normal limits	20	CS	39	3262	2	0 (2 developed new anterior compartment prolapse)
Rahmanou P. et al. [20]	1	40	3	−1	Sacrohysteropexy— Type 1 Polypropylene mesh	Uterine artery Dopplers at 23 weeks showed significant right angulation of both arteries at the level of the internal os but PI and RI remained within normal limits	11	CS	38	3290	3	0 (at 3 months, recurrence anterior compartment prolapse)
Pandeva I. et al. [21]	7	23–39 (range)	NA	NA	Sacrohysteropexy— Type 1 Polypropylene mesh	NA	6–36 (range)	CS	38	NA (normal weight)	6	1
Szymanowski et al. [22]	1	32	2	Stage II	Sacrohysteropexy—monofilament polyvinyl difluoride mesh	NA	20	CS	38	3220	10	0
Lewis C.M. et al. [23]	1	35	2	3	Sacrohysteropexy—lightweight polypropylene mesh	NA	15	CS	At term	4080	12	0 (at 2 years, recurrence anterior and posterior compartment prolapse)
Gadonneix P. et al. [24]	1	38	2	Stage II	Sacrohysteropexy—shaped Polyester mesh	NA	22	CS	38	NA	48	0
Albowitz M. et al. [25]	1	33	3	3.5	Sacrohysteropexy— Polypropylene, Poliglecaprone 25 mesh	NA	12	CS	38	NA	3	0
Pilka R. et al. [26]	1	38	3	Stage III	Sacrohysteropexy—polypropylene mesh	NA	12	CS	37	NA	18	0
Algeri et al. [27]	1	33	2	Stage II	Sacrohysteropexy—polypropylene mesh	NA	12	CS	35—for worsening pelvic pain	NA	NA	0
Samantray S.R. [28]	1	27	1	3	Sacrohysteropexy— Type 1 Polypropylene mesh	Normal	21	CS	38	3400	12	0
Rotem R. et al. [29]	4	NA	NA	NA	Sacrohysteropexy—lightweight polypropylene mesh	NA	NA	CS	NA	NA	65	0
Busby G. [30]	1	31	2	Stage II	Sacrohysteropexy—polypropylene mesh	NA	12	CS	36—for worsening pelvic pain	3140	2	0
Futcher F. et al. [31]	1	39	7	6	Sacrohysteropexy— 2 polypropylene mesh (attached to the cervix and together through the cervix)	NA	11	CS	39—gestational diabetes, foetus in breech position	3820	2	0

## Supplementary Materials

The following supporting information can be downloaded at: https://www.mdpi.com/article/10.3390/jcm14082777/s1, Table S1: JBI Critical Appraisal Checklist.

## Abbreviations

The following abbreviations are used in this manuscript:

POP	Pelvic Organ Prolapse
BMI	Body Mass Index
POP-Q	Pelvic Organ Prolapse Quantification
PI	Pulsatility Index
RI	Resistance Index
C-section	Caesarean-section
LSHP	Laparoscopic sacral hysteropexy
LHP	Laparoscopic hysteropectopexy
LLS	Laparoscopic lateral suspension
QOL	Quality of life
NA	Not applicable
