# Supplementary material for: Pregnancy After Laparoscopic Hysteropexy: A Systematic Review
Source: J Clin Med. 2025 Apr 17;14(8):2777. doi: 10.3390/jcm14082777 (PMC12027503; doi:10.3390/jcm14082777)
Supplement: Supplementary file 1 [file jcm-14-02777-s001.zip › jcm-3580163-supplementary.pdf]

Title	Author, Year	D1	D2	D3	D4	D5	D6	D7	D8
Pregnancy following laparoscopic hysteropexy—a case series	Jefferis H. Et al 2017	YES	YES	YES	YES	YES	YES	YES	YES
Pregnancy post-laparoscopic hysteropexy	Rahmanou P. Et al.	YES	YES	YES	YES	YES	YES	YES	YES
Efficacy and Pregnancy Outcomes of Laparoscopic Single Sheet Mesh Sacrohysteropexy	Pandeva I. Et al 2016	UNCLEAR	YES	UNCLEAR	YES	YES	YES	YES	YES
Cesarean section after laparoscopic hysterosacropexy with Richardson's lateral repair and Burch operation—Case report	Szymanowski et al. 2019	YES	YES	YES	YES	YES	YES	YES	YES
Sacrohysteropexy followed by successful pregnancy and eventual reoperation for prolapse	Lewis C.M. et al 2012	YES	YES	YES	YES	YES	YES	YES	YES
Pregnancy after laparoscopic sacral colpopexy: a case report	Gadonneix P. Et al. 2012	YES	YES	YES	YES	YES	YES	YES	YES
Pregnancy After a Laparoscopic Sacrohysteropexy : a Case Report	Albowitz M. Et al. 2014	YES	YES	YES	YES	YES	YES	YES	YES

Title	Author, Year	D1	D2	D3	D4	D5	D6	D7	D8
Laparoscopic hysterosacropexy with subsequent pregnancy and delivery by cesarean section: case report with short term follow-up.	Pilka R. Et al. 2019	YES	YES	YES	YES	YES	YES	YES	YES
Laparoscopic Hysterosacropexy : Is it A Safe Option for Fertility Spearing?	Algeri et al. 2023	YES	UNCLEAR	YES	YES	YES	YES	YES	YES
Successful Pregnancy Outcome After Laparoscopic Sacrohysteropexy for Pelvic Organ Prolapse	Samatray S.R. 2021	YES	YES	YES	YES	YES	YES	YES	YES
Long-term Outcomes of Laparoscopic Sacrohysteropexy with SERATEX® SlimSling® Mesh: A Retrospective Case Series	Rotem R. Et al. 2024	UNCLEAR	UNCLEAR	NO	YES	UNCLEAR	YES	YES	YES
Successful pregnancy outcome following laparoscopic sacrohysteropexy for second degree uterine prolapse	Busby G. et al 2010	YES	YES	YES	YES	YES	YES	YES	YES
Pregnancy after laparoscopic hysteropexy: Is there a prolapse recurrence?	Futcher F. et al 2023	YES	YES	YES	YES	YES	YES	YES	YES
